# Life Cycle Assessment of Cement Production with Marble Waste Sludges

**DOI:** 10.3390/ijerph182010968

**Published:** 2021-10-19

**Authors:** Antonio Ruiz Sánchez, Ventura Castillo Ramos, Manuel Sánchez Polo, María Victoria López Ramón, José Rivera Utrilla

**Affiliations:** 1 Department of Structure Mechanics and Hydraulic Engineering, University of Granada, 18071 Granada, Spain; 2 Department of Inorganic Chemistry, Faculty of Science, University of Granada, 18071 Granada, Spain; mansanch@ugr.es (M.S.P.); jrivera@ugr.es (J.R.U.); 3 Department of Inorganic and Organic Chemistry, Faculty of Experimental Science, University of Jaén, 23071 Jaén, Spain; mvlro@ujaen.es

**Keywords:** marble waste sludge, life cycle assessment (LCA), CO_2_, emissions, greenhouse gases (GHG), cement, limestone, environment

## Abstract

The construction industry has a considerable environmental impact in societies, which must be controlled to achieve adequate sustainability levels. In particular, cement production contributes 5–8% of CO_2_ emissions worldwide, mainly from the utilization of clinker. This study applied Life Cycle Assessment (LCA) methodology to investigate the environmental impact of cement production and explore environmental improvements obtained by adding marble waste sludges in the manufacture of Portland cement. It was considered that 6–35% of the limestone required for its production could be supplied by marble waste sludge (mainly calcite), meeting the EN 197-1:2011 norm. Energy consumption and greenhouse gas (GHG) emission data were obtained from the Ecovent database using commercial LCA software. All life cycle impact assessment indicators were lower for the proposed “eco-cement” than for conventional cement, attributable to changes in the utilization of limestone and clinker. The most favorable results were achieved when marble waste sludge completely replaced limestone and was added to clinker at 35%. In comparison to conventional Portland cement production, this process reduced GHG emissions by 34%, the use of turbine waters by 60%, and the emission of particles into the atmosphere by 50%. Application of LCA methodology allowed evaluation of the environmental impact and improvements obtained with the production of a type of functional eco-cement. This approach is indispensable for evaluating the environmental benefits of using marble waste sludges in the production of cement.

## 1. Introduction

Worldwide cement production has remained constant since the crisis in 2008, reaching 4100 million metric tons in 2019 [1] (Figure S1). According to the latest International Energy Agency report, global cement production is expected to be 12% higher by 2050, with an increase of 4% in direct CO_2_ emissions [2].

The cement industry has been reported to contribute 5–8% of global CO_2_ emissions [3,4], estimated at 1.50 ± 0.12 gigatons of CO_2_ in 2018 [5]. The calcination process contributes half of the CO_2_ emitted [6,7], with the emission of 850 kg CO_2_ per ton of clinker produced [8].

The CO_2_ emitted during clinker production derives from the combustion of fossil fuels to generate thermal energy and includes CO_2_ from the decomposition of CaCO_3_ into CaO and from the calcination process (limestone decarbonation) [9]. The latest update of the Cement Sustainability Initiative (CSI) database estimates the emission of 836 kg·CO_2_/t clinker in 2018, based on data from 21% of the world’s cement plants, lower than the rate of 844 kg·CO_2_/t clinker recorded in 2015 [10]. It was reported that 12,700,000 tons of clinker were produced in Spain in 2018, based on data from 63% of cement production plants, with the emission of 11,100,000 tons of CO_2_ [11]. Indirect CO_2_ emissions attributable to cement production result from the production of electric energy and from transportation and logistics.

According to Schneider [12], the greatest potential CO_2_ reduction potential can be achieved by replacing clinker with supplementary cementitious materials that deliver the appropriate performance and durability, which need to be readily available. Efforts to reduce CO_2_ emissions from cement production should focus not only on the calcination process but also on the processes responsible for the other 50% of emissions. Account should also be taken of the impact on the consumption of raw materials (limestone, clay, etc.), with 1.7 tons being needed to produce 1 ton of clinker [8]. The substitution of 40% of the clinker used in cement production could theoretically reduce the annual global emission of CO_2_ by up to 400 million tons.

In the 2015 Paris Agreement, the European Union (EU) committed to a 40% reduction in greenhouse gas (GHG) emissions versus 1990 levels by 2030 [13]. The European cement industry is governed by Directive 2003/87/CE, which assigns maximum CO_2_ emissions by sector and calculation method (emission rights) and sets out the instruments available to comply with the reductions agreed by each member State.

Potential environmental improvements investigated include reductions in the consumption of fossil fuels and raw materials, atmospheric emissions, effluent discharges, and solid waste associated with conventional Portland cement production.

Researchers have frequently characterized and studied the properties of cements and mortars with marble waste sludge over the past few years [14,15,16,17,18,19,20,21,22,23]. However, they have centered on the fulfillment of norms for mechanical composition and resistance (AENOR, 2005; AENOR, 2011) and have not addressed the issue of marble waste utilization from the perspective of environmental improvements. Some have referred to reductions in the carbon footprint achieved with additives [24,25] through a lesser consumption of cement clinker, but they have not presented the environmental argument.

Life cycle assessment (LCA) methodology was internationally formalized and standardized within the family of ISO 14,040 standards in the 1990s, with a broad revision in 2006. It is widely accepted by researchers and institutions and used by public administrations to formulate policies [26]. LCA offers the quantification of environmental pressures related to goods and services throughout their life cycle, including the acquisition, treatment, configuration, production, and use of raw materials and their recycling or final disposal, i.e., “from the cradle to the grave”.

The regulations establish four phases of LCA development: (i) definition of the objective and scope, including limits of the system and levels of detail; (ii) life cycle inventory (LCI) analysis, compiling and quantifying inputs and outputs throughout the life cycle of the product [27,28]; (iii) life cycle impact assessment (LCIA), determining and evaluating the magnitude and significance of the potential environmental impact of the product [29]; and (iv) interpretation of LCI and/or/LCIA findings, establishing conclusions and making recommendations.

Besides LCA, other environmental management techniques include risk evaluation, environmental performance evaluation, system dynamics, environmental audit, ecological footprint, GHG protocol, and LCIA. LCA is considered to provide the optimal framework for evaluating the potential environmental effects of products, and it has been used in research on waste, demonstrating that its environmental impact is a key question to be addressed [30,31].

Introduced by Jay Forrester in the 1960s, system dynamics is useful to understand complex large-scale management problems in accordance with the principles of systemic thinking [32]. The difference between LCA and system dynamics lies in their scope. Thus, LCA has a more limited scope, focusing on very specific environmental indicators, which should not be perceived as a drawback but rather as a mark of its specificity. An et al. [33] used LCA to compare various scenarios of cement production and CO_2_ capture. Although they found that some technological changes only minimally reduce the environmental impact of each ton of cement, they can have a major effect if applied throughout the cement sector, as noted by multiple researchers. The review by Wu et al. [34] concluded that LCA prevails over system dynamics. Nicoara et al. [35] evaluated the contribution of industrial waste, including marble powder, as supplementary cementitious material in cement manufacturing. After a wide review, they emphasized the importance of LCA alongside investigation of the mechanical and physicochemical properties of the material in order to establish the environmental feasibility of its utilization.

Table 1 lists the most recent investigations that used LCA methodology in relation to cement, mortar, and concrete products, either directly or within research on waste from construction and demolition. Few published studies have used LCA to assess the contribution of industrial additives or byproducts to cement and/or concrete, presented as complementary to technical evaluations. The table shows wide variations in the utilization and applicability of LCA among researchers. The method is frequently adapted to the aims of the investigation, which is a valid approach, although its principles, requirements, and guidelines have sometimes been followed in a rather relaxed manner. The review by Gursel et al. [27] highlights the need to continue quantitative research on the utilization of industrial additives and byproducts in concrete production, while Brito and Kurda 2021 [23] reviewed a series of potential strategies to reduce the negative impact of cement-based materials production.

The Global Warming Potential (GWP) of these processes is frequently evaluated in the aforementioned studies. It has been estimated that Portland cement production generates an average of 842 kg·CO_2_/ton of clinker produced and that around 6–7% of total anthropogenic GHG emissions derive from cement production. In order to achieve the agreed objective of a 50% reduction in total CO_2_ emissions by 2050, CO_2_ emissions from the cement industry need to be reduced by at least 18% [36].

The present study addresses the reduction of CO_2_ emissions in cement manufacturing by the utilization of marble waste byproducts and is therefore within the framework of Mechanisms of Clean Development, alongside different publications from a business management perspective [25]. LCA methodology is used in the present manuscript to evaluate environmental improvements in conventional Portland cement manufacturing that can be achieved by introducing marble waste sludge into its production. Account is also taken of the environmental benefit of eliminating marble waste from the mining industry, which is currently deposited in decantation pools as inert material, representing an environmental hazard.

**Table 1:** Applications of LCA for materials used in the production of cement, mortar, and concrete.

Material	Parameters Analyzed *	GHG **	Functional Unit	Stages Considered	Ref.
Cement production in Spain	ADP, GWP, ODP, HTP, TETP, POCP, AP, EP, MEP, LUP	21.6%	1 ton of grey cement	Cement production. CO_2_ capture	[37]
Cement with cementitious powder waste	GWP	Variable	-	Cement production	[38]
Cement production in China	GWP, AP, EP, POCP, HTP	-	1 ton of cement and with 42.5 MPa	Material acquisition. Processing and transportation to plant. Cement production	[39]
Substitution of cement in concrete with supplementary cementing materials	GWP	Variable	1 m^3^ of concrete	Material acquisition. Transportation to plant. Concrete production. Final transportation	[40]
Mixtures of concrete with blast furnace fly ash and slag	GWP	32% 62%	1 m^3^ of concrete	Material acquisition. Transportation to plant. Concrete production. Final transportation	[41]
Self-compacting concrete	GWP	-	1 m^3^ of premixed concrete	Premixed	[42]
Cement with additives	GWP, EC	12%	1 ton of cement	Material acquisition. Processing and transportation to plant. Cement production.	[43]
Construction product recycling	GWP	-	Cement production demand. Reference flow	Landfill. Downstream recycling. Recycling. Recycling after selective demolition	[44]
Concrete with ash from wastewater treatment plant sludges	ADP, GWP, ODP, HTP, TETP, POCP, AP, EP	9%	1 m^3^ of premixed concrete	Transportation and premixing	[45]
Cement with granite sludges	EC	-	-	Test tube preparation	[46]
Cement mortars with plastic waste and carbon fibers	GWP, EC	13.69%	1 m^3^ of cement paste	Cement production	[47]
Self-compacting concrete reducing binding material	GWP	16%	1 m^3^ of premixed concrete	Concrete production	[48]
Cement mortar with glass powder	GWP, ODP, AP, EP, POCP	20%	100 bags of cement	Cement production	[49]
Concrete reducing cement, adding metakaolin and steel fibers	GWP	Variable	1 m^3^ of premixed concrete	Obtaining raw material Fresh concrete production. Transportation	[50]
Ornamental stone waste added to cement	GWP, EC	9%	1 kg of product	Additive drying. Cement production. Transportation	[51]

* ADP: antibiotic depletion potential. GWP: global warming potential. ODP: ozone layer depletion potential, R11–CCl3 F. HTP: human toxicity potential, DCB–1,4- dichlorobenzene. TETP: terrestrial ecotoxicity potential. POCP: Photochemical ozone creation potential. AP: Acidification potential. EC: Energy consumption. EP: Eutrophication potential. MEP: Marine Eutrophication potential. LUP: Land-use potential. ** GHG = greenhouse gas emissions. The researchers use different terms for the same concept, e.g.,: KgCO_2_, carbon footprint, climate change, GWP, etc.

## 2. Materials and Methods

### 2.1. Marble Waste Sludges

The waste used in this study is sludge from the marble processing industry in the province of Almeria (Spain). It derives from the refrigeration process applied in the cutting and polishing of marble blocks and slabs, with the water being extracted by centrifugation and the remaining sludge deposited in a public decantation pool. The sludge is an inert waste, largely comprising calcite, with alkaline pH and particle size <1 μm. Sludge samples were gathered at a distance of 100 km from the factory where the clinker and cement was prepared. The technological validity of this proposal was examined by using the sludge samples to prepare six CEM II cements that fulfill European norm EN 197-1, increasing the proportion of sludge and reducing the percentage of clinker. Studies have confirmed the viability of this approach for the production of mixed cement [52]. The simultaneous adoption of these strategies in the concrete industry would reduce its environmental impact.

### 2.2. Study Strategies for Reducing Environmental Impacts

**Figure 1:**
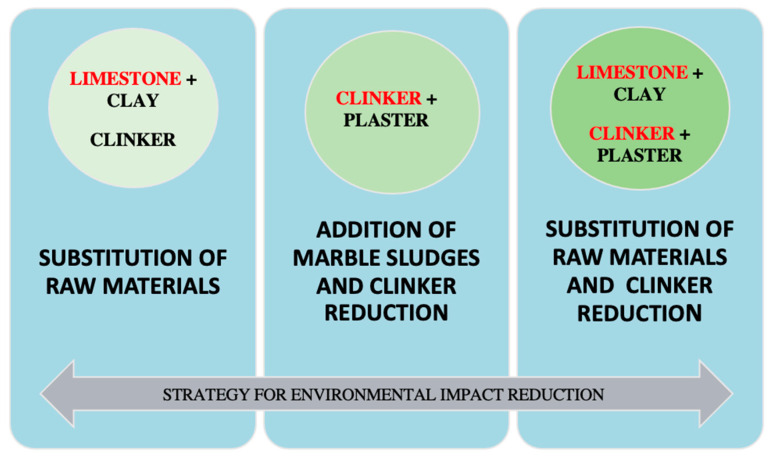
Diagram of the strategy to reduce the environmental impact of the cement industry by using carbonated sludge waste.

This study proposes three strategies to reduce the environmental impact of the cement industry based on the utilization of alternative raw materials and the application of LCA methodology, as described below and summarized in Figure 1:

- Utilization of carbonated sludge as substitute for raw materials used to produce cement clinker, maintaining composition percentages.
- Utilization of carbonated sludge as additive, reducing the percentage clinker in cement (by weight) to obtain CEM-II cement.
- Utilization of carbonated sludge to replace the raw materials that form the clinker, obtaining CEM-II cements with different proportions of carbonated sludge.

We have found no other published environmental and cost-benefit analyses of the use of waste powders in large-scale production, as also reported by other authors [53]. Besides replacing limestone with marble waste powder, other stages of conventional Portland cement manufacturing are affected by the present proposal, including the obtaining and transformation of raw materials and the preparation of the raw cement and cement. Schneider et al. [54] described different strategies for promoting sustainability in the cement industry, including the utilization of alternative fuels and raw materials. The present study focuses on the substitution of raw materials and the reduction in clinker.

### 2.3. Scope, System Boundary, and LCA Methodology

#### 2.3.1. Scope of the Study

The present study aims to assess and compare the environmental impact of Portland cement production and the derived “eco-cement” produced with marble wastes, applying LCA methodology focusing on the category of environmental impact of Climate Change or Global Warming Potential (GWP).

#### 2.3.2. Functional Unit

The functional unit is defined as 1 kg of cement at the factory gate. This study adopts a “cradle-to-gate” approach, so that the inventory includes processes associated with the production of these services. Sludge is generated in a process that classifies it as waste and does not therefore take account of its environmental burden. The problem with adopting a “cradle-to-grave” or “cradle-to-cradle” perspective is the lack of representative data related to the phases of use and end of useful life. The database for the life cycle inventory in the construction industry includes cement, aggregates, and water as examples of primary input materials, but it does not specifically include data on supplementary cementitious materials or recycled waste [55].

The setting of the functional unit is established, defining the quantification of the identified functions (performance characteristics) of the product [56]. This is important because it limits the concepts considered and quantified, it provides input and output data for the process, and, more importantly, it allows comparisons of LCA results among processes or products with the same functional performance. ISO 14,040 standard and 14,044 methodology were used for the purposes of this evaluation, which aimed to establish a framework rather than detailed guidelines. LCA studies need to use the same parameters for their functional units to permit their comparison. In this way, the ISO standard organizes the LCA into four main phases:

- Definition of the objectives and scope, making key decisions on the configuration and definition of the system under study.
- Inventory analysis, identifying and quantifying the energy, water, and materials used and environmental emissions, including solid waste, gas emissions, and wastewater discharge.
- Evaluation of the LCIA to identify and assess the amount and importance of potential environmental impacts. Inputs and outputs are first assigned to impact categories, and their potential impacts are quantified according to characterization factors. This step provides details on the indicators resulting from all impact categories; the importance of each impact category is evaluated by normalization and, finally, by weighting.
- The last phase comprises the interpretation and review of results, determination of data sensitivity, and presentation of conclusions.

#### 2.3.3. System Boundary

**Figure 2:**
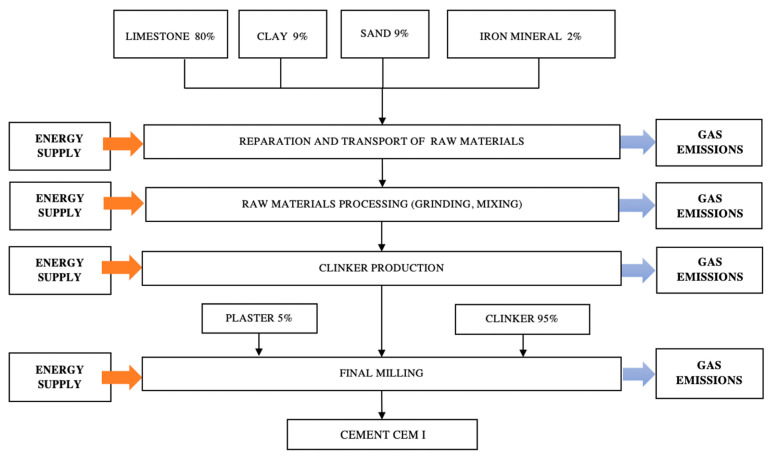
Basic process. Definition of the “cradle-to-gate” scope. System limits.

Figure 2 summarizes the system limits, including all of the supplies necessary for cement production. They cover the extraction of each raw material, its preparation and homogenization for producing raw cement, and its transportation. The raw cement is burned to obtain clinker, followed by milling and the addition of plaster. The final product is stored in bulk in the plant facilities.

Industrial machinery and equipment are not considered, because of the difficulty of inventorying all goods involved and because the environmental impact per product unit is considered low in the LCA framework in comparison to the other processes, being used over a prolonged time period and also in other processes.

The Portland cement production process and associated norms need to be summarized, taking EN 197-1 regulation as reference, in order to develop a strategy to reduce the environmental impact by using marble waste in powder form.

The process is divided into four phases (Figure 2): (I) Preparation and transport of raw materials, obtaining the limestone, clay, sand, and iron mineral in the quarry, grinding them, and then transporting them to the plant; (II) Raw material processing, homogenizing components by selective grinding and mixing, producing raw cement; (III) Clinker production; burning the raw cement, which is usually preheated; and (IV) Milling of clinker and additives; after cooling, plaster and other additives are added to the clinker, and the resulting mixture is milled. The final product, ordinary Portland cement, is then stored for subsequent distribution.

Finally, it should also be considered that the system suggested is for full-scale production and that cement is an essential component of two products heavily used in construction worldwide, i.e., concrete and mortar. Some studies report that Portland cement is the main source of CO_2_ emissions in concrete mixtures, reaching 81% of total CO_2_ emissions [57], and concrete has become the second most widely used substance in the world after water [58].

The designations of ordinary Portland cements and those with additives are listed in supplementary information (Table S1).

#### 2.3.4. Life Cycle Inventory Analysis and Impact Assessment

LCA methodology requires definition of the environmental impact categories in cement production, which are GWP and primary energy demand. The former is quantified as kg of equivalent CO_2_, using the global protocol for Community-scale GHG emissions (GPC) of the international plant protection convention (IPPC) [59], while the latter is expressed as cumulative energy demand (CED) in MJ-equivalents. The CED of a product represents the direct and indirect energy consumption over its life cycle, including the energy consumed during the extraction, manufacturing, and removal of raw and auxiliary materials [60]. It has been reported that the main environmental burden of concrete production and the highest GHG emissions over its life cycle result from the manufacture of cement [61]. It has been proposed that the combined utilization of waste fuels and cements with additives alongside technological improvements in energy efficiency could reduce GHG emissions from cement manufacturing by 11% [6,62]. The addition of limestone to Portland cement (during concrete production) has been estimated to reduce GHG emissions by 4% [63]. On the other hand, it has been found that improvements in transportation and the end-of-life of processes have relatively little impact on global GHG emissions from cement and concrete manufacturing, reducing them by less than 2% [64].

Emissions to the atmosphere of the clinker production system largely depend on the system design and on the nature and composition of the raw materials and fuels [65]. The base scheme of this research was the synthesis of processes performed in the reference cement factory (Figure 2), which uses a dry process. Based on the contribution of inputs and emissions, we obtained four stages: supply and preparation of raw materials; mixing and homogenization; clinkering; and grinding and mixing of clinker and additive.

The study hypothesis was that negative environmental impacts will be reduced when larger amounts of marble waste powder sludge are used to replace limestone in the production of raw cement and as a replacement for cement clinker. As observed in Table 2, the composition of the mixture follows Spanish norms for the reception of cement (RC-16) [66]. The percentage of plaster, used as setting retardant, remains constant and within the limit established by the EN 197-1 norm for sulfate (SO_3_) content (<3.5% of the final cement weight).

The primary data on energy consumption and GHG emissions during marble cutting and cement transportation and manufacturing were complemented with average data from Europe in the Ecoinvent V3.7, Industry data library v.2017, Agri-footprint v5.0, US Life Cycle Inventory Database v2021, European and Danish Input/Output database, Environmental Footprint (EF.v2.0) and EXIOBASE v3.3 databases, using the commercial software SimaPro LCA version 9.1.1 (PRé Sustainability, Amersfoort, Netherlands), one of the most widely accepted methodologies in Europe [67].

The impacts were assessed by SimaPro version 9.1.1 software using the ReCiPe2016 method, since it provides harmonized implementation of the cause-and-effect pathways for the calculation of the characterization factors of the midpoint and the end point. This methodology is framed in the European level and is considered as the successor of previous methodologies (CML2001 and ECO-Indicator99). It integrates the approach oriented to the environmental problem and the approach oriented to the damage.

Life Cycle Impact Assessment (LCIA) translates emissions and resource extractions into a limited number of environmental impact results through the characterization factors. Each substance, resource and extraction belonging to the manufacturing process was classified and accounted for according to its group of resources, air, and soil compartments. Those groups are the emissions that contribute to the levels of toxicity of the manufacturing process. Its classification is based on the ISO 14,044 operational guide of the LCA manual and the SimaPro software was used for accounting.

The values of characterization factors for each issued substance are listed in the ACV manual: Operational Annex [68]. The calculations to perform the characterization, that is, to obtain the environmental impacts, were calculated with the SimaPro software.

## 3. Results

**Figure 3:**
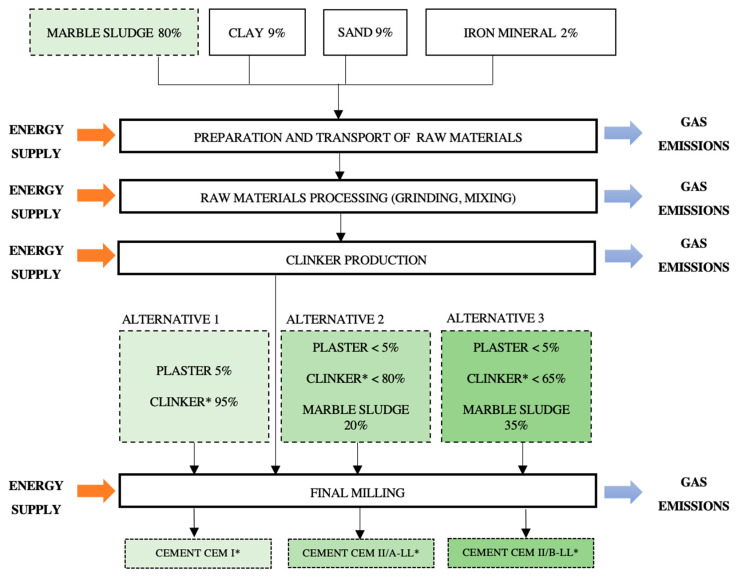
Process with limestone substitution in raw cement; process with limestone substitution in raw clinker with 20% additives; and process with limestone substitution in raw clinker with 35% additives. * The asterisk sign was used to differentiate the derived eco-cements from the original class of cements.

Among the scenarios examined in the LCA (Figure 3), the first is the total substitution of limestone by waste marble powder sludge to form raw cement that is then burned, yielding the Clinker* to produce cement CEM I* after the addition of plaster as retardant. This approach avoids the extraction of limestone from the quarry and its grinding, transportation, milling, and pre-homogenization. Given that the percentage CaCO_3_ is 98.52% for waste marble powder sludge versus 95% for limestone, a lower amount of waste is required to produce raw cement (0.698 kg/kg of waste vs. 0.705 kg/kg of limestone), as shown in Table 3. In addition, the waste is dried outdoors and requires no electricity to reduce its humidity. Emissions from this material in cement production are therefore considered negligible and are not included in the system limits, only taking account of emissions from its processing and transportation.

As part of the scenario of total limestone substitution in raw cement to obtain clinker*, two maximum percentages of waste marble powder sludge were used, in accordance with technical norm RC-16 (options 2 and 3, Figure 3), reducing the amount of clinker* required. In alternative 2, the waste is added with clinker* and plaster in the final milling phase, obtaining CEM II/A-LL* with a maximum addition of 20% waste and proportional reduction in clinker*. The letter A is included in this designation because the percentage addition is ≤20%, and LL because the total organic carbon content in the waste marble powder sludge is <0.2%.

LCA alternative 3, using the percentages permitted by EN 197-1, includes the highest percentage of waste marble powder sludge and should therefore deliver the greatest reduction in environmental impact. Limestone is replaced by the waste in the formation of raw cement and the production of clinker*, which is milled to produce cement CEM II/B-LL*, containing <65% clinker with plaster and 35% waste.

The inventory (Table 3) was based on data provided by the factory and complemented by the Ecoinvent database and published corrections [28]. High-quality data are essential for the evaluation of environmental performance, especially for comparative ratings [69].

Table 3 shows that the marble waste sludge has a higher purity (CaCO_3_ content of 98.52% vs. 95.00%), indicating that a smaller amount is required to substitute limestone, although its CO_2_ emissions are slightly higher (0.008 kg/kg). One advantage of using marble waste sludge is that it does not require grinding, and its mixture with clinker requires much less energy to prepare raw cement, with 75% less energy being used in the milling process (0.012 kWh/kg vs. 0.048 kWh/kg). Finally, total material transportation costs are lower when limestone is replaced with waste sludge, which is taken to the factory by truck, with no transportation by boat.

The most relevant factor explaining the more favorable environmental impact of preparing eco-cement CEM II/B-LL* is the lesser amount of clinker consumed when marble waste sludge is added. Given that CEM I is an additive-free cement, the truck transportation it requires is half that needed for CEM II/B-LL*.

After carrying out the inventory, an LCIA was performed, assigning inventory inputs and outputs to the different impact categories. Based on the characterization factors, indicators were obtained for the conventional process and for the process using 35% additives with total limestone substitution, as the environmental strategy with greatest impact (Table 4).

**Figure 4:**
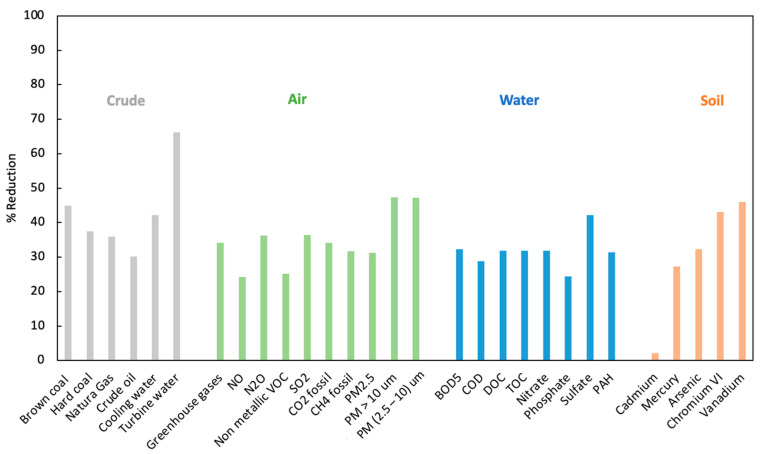
Chart showing lower LCIA indicators for the process with limestone substitution and 35% additives (alternative 3) than for the basic process.

The final LCA phase is the interpretation of LCIA results, highlighting the values of the most relevant substances (Figure 4). All of these values are lower in the eco-cement proposal than in conventional production because of the action taken on limestone and clinker contents. Given that limestone is a primary element in cement production, it has a higher limestone kg/clinker kg ratio than the products that comprise it. In the case of clinker, reducing the amount necessary to produce 1 kg of cement directly and proportionally yields a reduction in supplies and emissions.

Importantly, the LCIA reveals that GHG levels are around 34% lower for the production of CEM II/B-LL* versus CEM I cement. In addition, 60% less water is used for turbines and a lower amount of particles is emitted into the air. Future LCA studies of eco-cement or “green concrete” should address specific functional concrete measures and exposure conditions expected [70]. Likewise, an aspect of increasing scientific interest in LCA analyses is the need to model rebound or recovery effects [71], given that the environmental impact of efficiency measures is not necessarily in the same direction, sometimes producing indirect effects that are not always positive.

## 4. Conclusions

This study uses LCA methodology to present an example of sustainability improvement in industry. It was applied to evaluate a reduction in the environmental impact of cement production. The results allow a priori assessment by industries of the effects of adding marble powder in cement production.

All LCIA indicators are lower for the proposed eco-cement than for conventionally produced cement. The actions on limestone and clinker, two key elements in cement production, directly and proportionally reduce inputs and emissions.

GHG emissions are around 34% lower with the production of CEM II/B-LL* cement than with the production of CEM I cement.

The replacement of limestone with marble powder sludge in raw cement avoids three stages in the production process: extraction, transportation from the quarry, and roll milling. The addition of marble sludge to clinker in cement production avoids ball milling, reducing GHG emissions and energy consumption. The higher percentage addition of sledge, the less clinker is required, which also reduces the GHG emitted in the clinkering process.

The system limits considered in this study covers the extraction of raw materials, preparation and production of cement and transportation. However, industrial machinery and equipment might be considered for a more in detailed future LCA analysis, even though they are involved and shared with other industrial processes. An analysis of LCA for eco-cement production is given, considering Spanish regional set-up following national and European standards and norms. Nevertheless, it could be generalized and extended to other similar processes adopting their constrains, specifications, and regulations.

It is worth mentioning that, for example, one of the strategies in the present study is to replace 6–35% of the limestone with marble waste powder in “green” cements, obtaining a product that meets European Norm EN 197-1:2011. It is not a question of merely presenting an option but rather evaluating the technological validity of its real-world application in the market, given that the scientific literature has long addressed the possibility of using limestone additives for cement and concrete.

It would be also interesting to analyze model rebounds or recovery effects given that the environmental impact of efficiency measures is not necessarily in the same direction, sometimes producing indirect effects that are not always positive. In addition, Future LCA studies of eco-cement or “green concrete” should address specific functional concrete measures and exposure conditions expected.

## Figures and Tables

**Table 2:** Study of cases as a function of the common cement classification of the norm for the reception of cements (RC-16) and the contribution of marble waste powder.

**Materials**	**Type of Cement (RC-16)**		
	CEM I	CEM II/A-LL	CEM II/B-LL
Clinker	95–100%	80–94%	65–79%
Plaster	<5%	<5%	<5%
Limestone	-	<20%	<35%
**Materials**	**Type of Cement**		
	CEM I *	CEM II/A-LL *	CEM II/B-LL *
Clinker	95–100%	80–94%	65–79%
Plaster	<5%	<5%	<5%
Carbonated sludge	80% of raw cement	80% of raw + <20%	80% of raw + <35%

* The asterisk sign was used to differentiate the derived eco-cements from the original class of cements.

**Table 3:** Basic inventory and process with limestone substitution and 35% additives.

Component	Basic Process	Limestone Substitution and 35% Additives	Data Source
	Clinker	Clinker *	
Coal	4.90 × 10^−2^ kg/kg	4.90 × 10^−2^ kg/kg	Factory
Petroleum coke	6.50 × 10^−2^ kg/kg	6.50 × 10^−2^ kg/kg	Factory
Fuel oil	0.0122 kg/kg	0.0122 kg/kg	Factory
Diesel	0.001 kg/kg	0.001 kg/kg	Factory
Natural gas	1.06 × 10^−4^ MJ/kg	1.06 × 10^−4^ MJ/kg	Factory
CO_2_ from fuels	0.390 kg/kg	0.390 kg/kg	Ecoinvent
CO_2_ from limestone	0.295 kg/kg	0.303 kg/kg	Ecoinvent
Slag	0.213 kg/kg	0.218 kg/kg	Factory
Sandstone	0.071 kg/kg	0.072 kg/kg	Factory
Lamellae	0.012 kg/kg	0.012 kg/kg	Factory
Limestone/Marble waste sludge powder	0.705 kg/kg	0.698 kg/kg	Factory
EE in clinker	0.0476 KWh/kg	0.0476 KWh/kg	Ecoinvent
EE in raw cement milling	0.048 KWh/kg	0.012 KWh/kg	Ecoinvent
Process water	0.00059 m^3^/kg	0.00059 m^3^/kg	Factory
Drinking water	0.000447 ton/kg	0.000447 ton/kg	Factory
Transport by truck	0.188 tkm/kg	0.224 tkm/kg	Ecoinvent
Transport by boat	0.17 tkm/kg	-	Ecoinvent
**CEMENT**	**CEM I**	CEM II/B-LL *	
Transport by truck	0.0606 tkm/kg	0.1225 tkm/kg	Ecoinvent
Total clinker consumed	0.950 kg/kg	0.603 kg/kg	Factory
Plaster	0.050 kg/kg	0.041 kg/kg	Factory
Marble waste sludge powder	0 kg/kg	0.355 kg/kg	-
Electrical energy	0.052 KWh/kg	0.0338 KWh/kg	Ecoinvent

EE = Electrical energy. * The asterisk sign was used to differentiate the derived eco-cements from the original class of cements.

**Table 4:** Results of LCIA indicators, conventional process, and process with limestone substitution and 35% additives.

Substance	Section	Unit	Normal Process	Process with Limestone Substitution and 35% Additives
Coal, brown, in soil	Raw	kg	2.6018 × 10^−2^	1.4320 × 10^−2^
Coal, hard, not specified, in soil	Raw	kg	8.1269 × 10^−2^	5.0780 × 10^−2^
Gas, natural, in soil	Raw	m^3^	1.6506 × 10^−2^	1.0564 × 10^−2^
Oil, crude, in soil	Raw	kg	1.1691 × 10^−1^	8.1591 × 10^−2^
Water, cooling, natural origin not specified/m^3^	Raw	m^3^	3.1323 × 10^−3^	1.8084 × 10^−3^
Water, turbine use, natural origin not specified	Raw	m^3^	1.3589	4.5948 × 10^−1^
Carbon dioxide, fossil	Air	kg	8.4230 × 10^−1^	5.5511 × 10^−1^
Dinitrogen monoxide	Air	kg	7.0630 × 10^−6^	4.4983 × 10^−6^
Methane, fossil	Air	kg	4.7811 × 10^−4^	3.2678 × 10^−4^
GHG	Air	kg	8.5636 × 10^−1^	5.6462 × 10^−1^
Nitrogen oxide	Air	kg	1.5937 × 10^−3^	1.2070 × 10^−3^
NMVOC	Air	kg	2.9612 × 10^−4^	2.2172 × 10^−4^
Particles, <2.5 um	Air	kg	8.9508 × 10^−5^	6.1561 × 10^−5^
Particles, >10 um	Air	kg	3.5062 × 10^−4^	1.8458 × 10^−4^
Particles, >2.5 um and <10 um	Air	kg	1.0298 × 10^−4^	5.4322 × 10^−5^
Sulfur dioxide	Air	kg	3.3898 × 10^−3^	2.1530 × 10^−3^
BOD5, biological oxygen demand	Water	kg	1.3404 × 10^−3^	9.0632 × 10^−4^
COD, chemical oxygen demand	Water	kg	1.5508 × 10^−3^	1.1052 × 10^−3^
DOC, dissolved organic carbon	Water	kg	4.2541 × 10^−4^	2.8977 × 10^−4^
Nitrate	Water	kg	2.5444 × 10^−6^	1.7342 × 10^−6^
PAH (Polycyclic Aromatic Hydrocarbons)	Water	kg	3.9898 × 10^−8^	2.7401 × 10^−8^
Phosphate	Water	kg	5.2351 × 10^−6^	3.9545 × 10^−6^
Sulfate	Water	kg	4.9035 × 10^−4^	2.8335 × 10^−4^
TOC, total organic carbon	Water	kg	4.2604 × 10^−4^	2.9025 × 10^−4^
Arsenic	Soil	kg	1.2491 × 10^−9^	8.4610 × 10^−10^
Cadmium	Soil	kg	1.6440 × 10^−10^	1.6072 × 10^−10^
Chromium VI	Soil	kg	3.8321 × 10^−8^	2.1789 × 10^−8^
Mercury	Soil	kg	1.1348 × 10^−12^	8.2473 × 10^−13^
Nickel	Soil	kg	1.5667 × 10^−9^	1.7566 × 10^−9^
Vanadium	Soil	kg	1.5897 × 10^−10^	8.5736 × 10^−11^
Zinc	Soil	kg	3.3479 × 10^−7^	3.8050 × 10^−7^

## Supplementary Materials

The following are available online at https://www.mdpi.com/article/10.3390/ijerph182010968/s1, Figure S1: Cement production worldwide. Source: Statica©, Figure S2: Documents published for the study keywords during the period 1994–2020 in the Scopus© bibliographic database, Figure S3: Graphic representation of the distribution of documents with the key words of interest by thematic area during the period 1994–2020 in the Scopus© database, Table S1: Classification of common cements. Obtained from the Instruction of the Reception of Cements (RC-16).
