# Supplementary material for: Life Cycle Assessment of Cement Production with Marble Waste Sludges
Source: Int J Environ Res Public Health. 2021 Oct 19;18(20):10968. doi: 10.3390/ijerph182010968 (PMC8535264; doi:10.3390/ijerph182010968)
Supplement: Supplementary file 1 [file ijerph-18-10968-s001.zip › ijerph-1377796-supplementary.pdf]

International Journal of
Environmental Research and Public Health

Article

Life Cycle Assessment of Cement Production with Marble Waste Sludges

SUPPORTING INFORMATION

Antonio Ruiz Sánchez [1,*], Ventura Castillo Ramos [2,*], Manuel Sánchez Polo [2], María Victoria López Ramón [3] and José Rivera Utrilla [2]

[1] Affiliation 1; Department of Structure Mechanics and Hydraulic Engineering, University of Granada, 18071, Granada, Spain. antonioruiz@ugr.es
[2] Affiliation 2; Department of Inorganic Chemistry, Faculty of Science, University of Granada, 18071, Granada, Spain. vcastillo@ugr.es, mansanch@ugr.es, jrivera@ugr.es
[3] Affiliation 3; Department of Inorganic and Organic Chemistry, Faculty of Experimental Science, University of Jaén, 23071, Jaén, Spain. mvlro@ujaen.es
* Correspondence: antonioruiz@ugr.es, vcastillo@ugr.es

1. Cement production

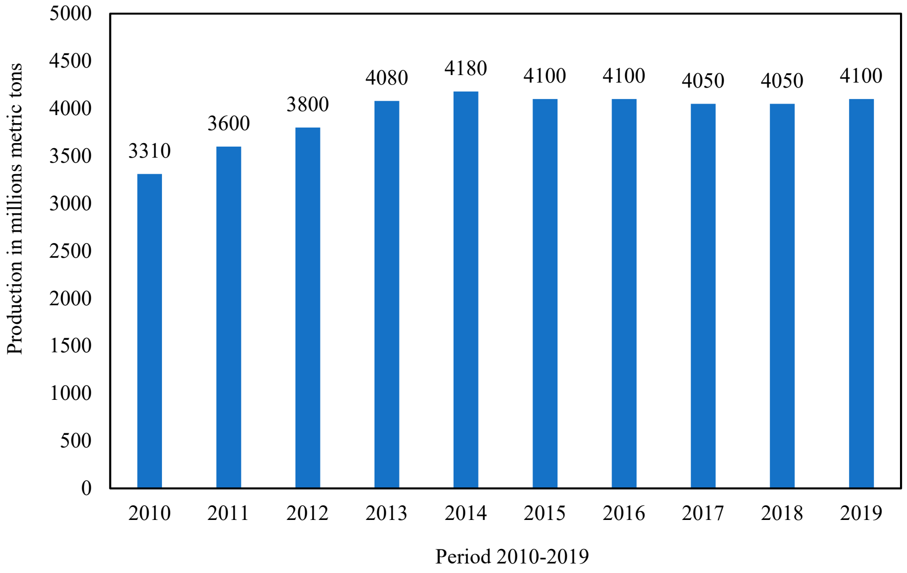

Figure S1. Cement production worldwide. Source: Statica©.

2. Research interest

Results of the search for key words "cement, waste, and marble" in the Scopus© bibliographic database (access date 5 November 2020) between 1994 and 2020 exemplifies the strong interest in the production of "green" cement by adding waste, specifically marble waste. Figure S2 depicts the major increase in the scientific literature with these study terms over the past few years, reflecting interest in the use of marble waste. This interest is also attributable to limitations established by some national governments on the exploitation of natural resources in riverbeds, considerably reducing access to natural sands and gravels for use in cement, mortars, and cement concretes. It is also connected to the increasing environmental limitations imposed on the cement industry.

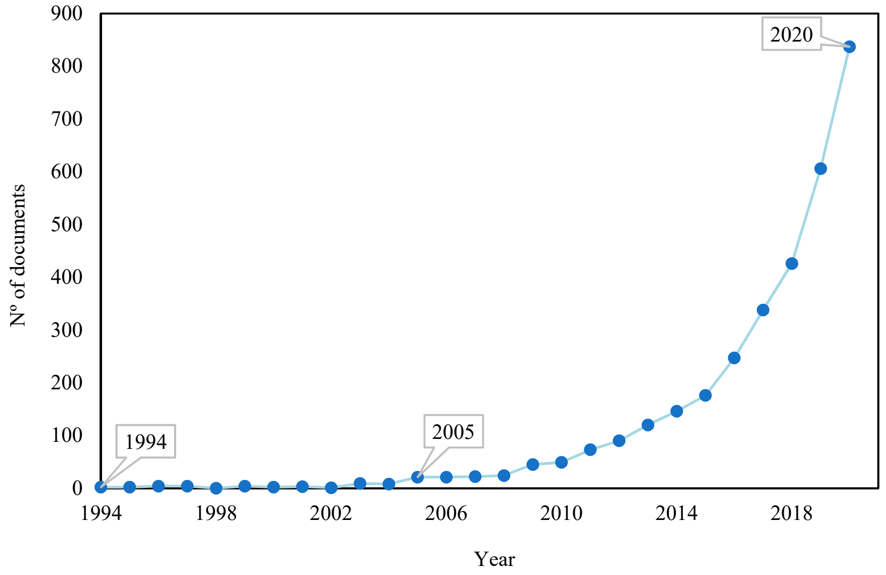

Figure S2. Documents published for the study keywords during the period 1994-2020 in the Scopus© bibliographic database.

Figure S3 shows that more than one-third of documents are referenced to Engineering (31%), 21% to Materials Science (21%), and only one-tenth to Environmental Science (11.9%). This indicates the need for greater in-depth study of the environmental effects of using marble waste in cement production.

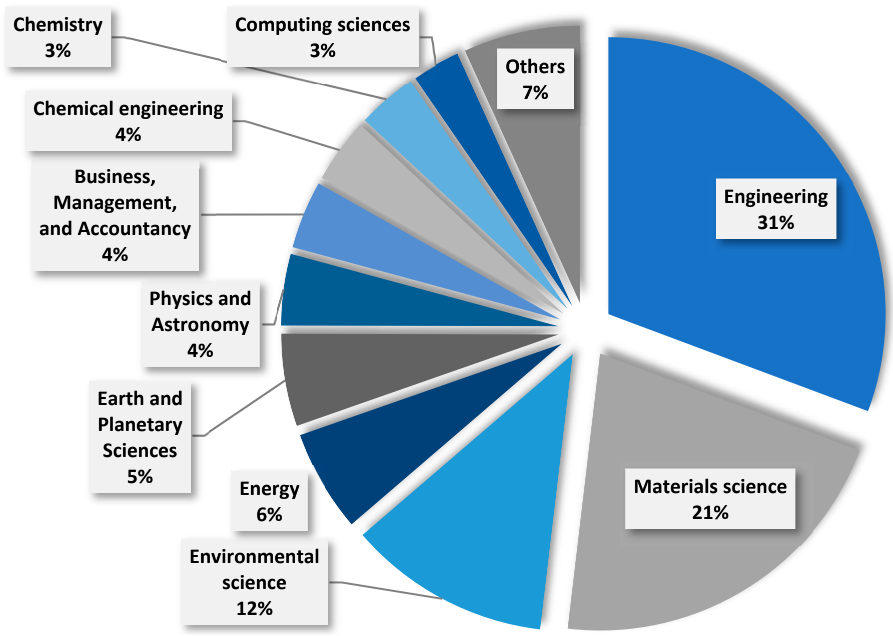

Figure S3. Graphic representation of the distribution of documents with the key words of interest by thematic area during the period 1994-2020 in the Scopus© database.

3. Cement nomenclature and classification

Common cements defined in norm UNE-EN 197-1 are gathered in Spanish regulations through the Instruction for the Reception of Cements RC-16 [1]. Table S1 compiles Instruction RC-16 on the nomenclature for ordinary Portland cement and Portland cement with additives. It can be observed that cement CEM I contains > 95% clinker by weight, with the remaining 5% being plaster as setting retardant. This is the maximum percentage of plaster in all of these cements. CEM II nomenclature is used to represent variations in percentage clinker and the emergence of additives other than plaster, designating cements as subtypes A or B according to the smaller or larger amount of additives used and adding a letter to identify the main component used as additive, as follows:

S: blast furnace slag
D: silica smoke
P: natural pozzolan
Q: calcined natural pozzolan
V: siliceous fly ash
W: calcareous fly ash
T: calcined shales
L: limestone with total organic carbon content ≤ 0.5% in mass
LL: limestone with total organic carbon content ≤ 0.2% in mas

International Journal of
Environmental Research and Public Health

MDPI

Table S1. Classification of common cements. Obtained from the Instruction of the Reception of Cements (RC-16)

TYPE	NOMENCLATURE	DESIGNATION	COMPOSITION (PROPORTION IN MASS)										
			MAIN COMPONENTS										MINOR COMPONENTS
			Clinker K	Blast furnace slag S	Silica smoke D	Puzzolan		Fly ash		Calcined shales T	Limestone		
						Natural P	Calcined natural Q	Siliceous V	Calcareous W		L	LL	
CEM I	Portland cement	CEM I	95-100	-	-	-	-	-	-	-	-	-	0-5
CEM II	Portland cement with slag	CEM II/A-S	80-94	6-20	-	-	-	-	-	-	-	-	0-5
		CEM II/B-S	65-79	21-35	-	-	-	-	-	-	-	-	0-5
	Portland cement with silica smoke	CEM II/A-D	90-94	-	6-10	-	-	-	-	-	-	-	0-5
	Portland cement with pozzolan	CEM II/A-P	80-94	-	-	6-20	-	-	-	-	-	-	0-5
		CEM II/B-P	65-79	-	-	21-35	-	-	-	-	-	-	0-5
		CEM II/A-Q	80-94	-	-	-	6-20	-	-	-	-	-	0-5
		CEM II/B-Q	65-79	-	-	-	21-35	-	-	-	-	-	0-5
	Portland cement with fly ash	CEM II/A-V	80-94	-	-	-	-	6-20	-	-	-	-	0-5
		CEM II/B-V	65-79	-	-	-	-	21-35	-	-	-	-	0-5
		CEM II/A-W	80-94	-	-	-	-	-	6-20	-	-	-	0-5
		CEM II/B-W	65-79	-	-	-	-	-	21-35	-	-	-	0-5
	Portland cement with calcined shales	CEM II/A-T	80-94	-	-	-	-	-	-	6-20	-	-	0-5
		CEM II/B-T	65-79	-	-	-	-	-	-	21-35	-	-	0-5
	Portland cement with limestone	CEM II/A-L	80-94	-	-	-	-	-	-	-	6-20	-	0-5
		CEM II/B-L	65-79	-	-	-	-	-	-	-	21-35	-	0-5
		CEM II/A-LL	80-94	-	-	-	-	-	-	-	-	6-20	0-5
		CEM II/B-LL	65-79	-	-	-	-	-	-	-	-	21-35	0-5
	Compound Portland cement	CEM II/A-M	80-88	12-20									0-5
		CEM II/B-M	65-79	21-35									0-5

International Journal of
Environmental Research and Public Health

Reference

[1] B. O. del E. Ministerio de la Presidencia, "Real Decreto 256/2016, de 10 de junio, por el que se aprueba la Instrucción para la recepción de cementos RC-16. 153: 45755-45824," 2016.
